# Epidemiology of COVID-19 outbreak in Ghana, 2020

**DOI:** 10.4314/gmj.v54i4s.3

**Published:** 2020-12

**Authors:** Ernest Kenu, Magdalene A Odikro, Keziah L Malm, Franklin Asiedu-Bekoe, Charles L Noora, Joseph A Frimpong, Benedict Calys-Tagoe, Kwadwo A Koram

**Affiliations:** 1 GFELTP, Department of Epidemiology and Disease Control, School of Public Health, University of Ghana, Legon, Accra, Ghana; 2 National Malaria Control Programme, Ghana Health Service, Accra, Ghana; 3 Disease Surveillance Department, Ghana Health Service, Accra, Ghana; 4 Department of Community Health, University of Ghana Medical School, Accra, Ghana; 5 Department of Epidemiology, Noguchi Memorial Institute for Medical Research, University of Ghana, Legon, Accra, Ghana

**Keywords:** COVID-19, Epidemiology, pandemic, Ghana, geospatial

## Abstract

**Objective:**

Describe the epidemiology of COVID-19 cases detected in the first four months of the pandemic in Ghana by person, place and time to provide an understanding of the local epidemiology of the disease.

**Methods:**

We conducted an exploratory descriptive study of all confirmed COVID-19 cases in Ghana from March 12 to June 30, 2020. Data was merged from the country's electronic databases, cleaned and summarized using medians, proportions and geospatial analysis.

**Design:**

A cross-sectional study design

**Setting:**

Ghana

**Participants:**

All confirmed COVID-19 cases in Ghana from March 12 to June 30, 2020

**Interventions:**

None

**Main Outcome measures:**

Epidemiological characterization of all confirmed COVID-19 cases recorded from March 12 – June 30, 2020 in Ghana by person, place and time.

**Results:**

A total of 17,763 cases were recorded with median age (IQR) of 33years (One month to 85 years). Among the confirmed cases, 10,272 (57.8%) were males and 3,521 (19.8%) were symptomatic with cough recorded in 1,420 (40.3%) cases. The remaining 14,242 (80.2%) were asymptomatic. Greater Accra region recorded the highest number of confirmed cases 11,348 (63.9%). All 16 administrative regions had recorded cases of COVID-19 by June 30, 2020 due to internal migration between the hotspots and other regions. The epidemiological curve showed a propagated outbreak with 117 deaths (CFR= 0.67%) recorded.

**Conclusion:**

A propagated outbreak of COVID – 19 was confirmed in Ghana on March 12, 2020. Internal migration from hotspots to other regions led to the spread of the virus across the nation. Majority of cases were asymptomatic.

**Funding:**

The COVID-19 pandemic response and writing workshop by the Ghana Field Epidemiology and Laboratory Training Programme (GFELTP) was supported with funding from President Malaria Initiative – CDC, and Korea International Cooperation Agency (on CDC CoAg 6NU2GGH001876) through AFENET

## Introduction

Coronaviruses are RNA viruses that cause respiratory, hepatic and neurological diseases in domestic and wild animals, and humans.^1^,^2^ Among humans, six species of coronavirus have been identified to cause disease. 3 Among these, Severe Acute Respiratory Syndrome (SARS-CoV) and Middle East Respiratory Syndrome (MERS-CoV) are of zoonotic origin and have been known to cause severe acute respiratory syndrome outbreaks among humans. 4–7 With increasing human to animal contact and interfaces in recent times, an outbreak of novel zoonotic species of Coronavirus was reported in late 2019 in Wuhan, China.^8^–^10^ The causative pathogen for the outbreak was identified as SARS - Coronavirus - 2 which rapidly spread through China and other parts of the world.^11^–^13^The disease was thereafter named COVID-19.

Symptoms of COVID-19 include fever, dry cough, headache, sore throat, cold, difficulty in breathing, muscle pain and malaise. 14,15 From large cohorts of patients, it was found that the disease presents as self-limiting in approximately 80% of infected individuals with 15% developing severe pneumonia and mortality estimated at 3.5%.^16^–^18^ Individuals with underlying health conditions and the aged are more susceptible to the infection and have higher chances of severe outcomes. 15,19,20

The World Health Organization (WHO) declared COVID-19 as a Public Health Emergency of International Concern (PHEIC) on January 30, 2020 and later as the number of cases and territories reporting cases increased globally, COVID-19 was declared a pandemic on March 11, 2020. 21 As at July 1^st^, 2020, over 213 countries were affected with over 10 million cases and 515,000 deaths. 22 Currently most of the countries affected have reported both imported cases and local transmission. Heightened surveillance, tracing of case contacts, varying degrees of restrictions on movement, preventive measures including physical distancing, wearing of nose mask and personal hygiene are among the measures taken by countries to halt the spread of COVID-19. 23–26

Ghana recorded its first two cases on March 12, 2020. These were imported from Norway and Turkey. The country began its response with heightened surveillance for identification of cases and subsequent tracing of their contacts. Mandatory quarantine of travelers entering the country was imposed on March 17, 2020. The borders were subsequently closed on March 22, 2020. As the number of cases continued to increase to a little above 60, restrictions were placed on movement in the Greater Accra and Ashanti Regions on March 30, 2020 as response continued. Voluntary testing of people within 1–2 km radius of identified cases was also instituted. With these response strategies, the country was able to detect about 1000 additional cases of which about 90% were asymptomatic. As at June 30, 2020, over 300,000 tests have been conducted with over 17,000 confirmed cases, 117 deaths and 13,000 recoveries. 27

We describe here the epidemiology of the outbreak in Ghana as at June 30, 2020 to provide valuable insights into the development of this pandemic and to guide the response as the disease continues to spread.

## Methods

### Study Design

We conducted a cross sectional descriptive exploratory analysis of all diagnosed COVID-19 cases in Ghana from March 12 to June 30, 2020.

### Study Setting

Ghana is a West African country occupying an area of 239,460 sq. km and lies between latitude 5° and 11° north. Ghana shares borders with Burkina Faso to the North, Togo to the east, Côte d'Ivoire to the west and the Atlantic Ocean to the south. The projected population from the 2010 national census as at May 2020 stands at 30,955,204. 28

The Ghana Health Service (GHS) is the largest public sector agency under the Ministry of Health that is responsible for provision of health service delivery to the Ghanaian population. Their mandate is to ensure that the health needs of Ghanaians are met. The country has five designated levels of health care starting from Community-based Health and Planning Services (level 1), health centers and clinics (level 2), district hospitals (level 3), regional hospitals (level 4) and tertiary hospitals (level 5). 29 Ghana has a well-structured health organogram with fairly adequate human resources and disease surveillance continues to improve across the country.

The two main epicenters of the country during the first four months of the COVID outbreak were Greater Accra and Ashanti Regions. Ashanti region is the third largest of the 16 administrative regions covering 10.2% of the total land area of Ghana. The region is the most densely populated in Ghana with an estimated 5,924,478 inhabitants according to the Ghana Statistical Service projected population for May 2020. 28 Greater Accra region occupies 1.4% of the total land area of the country. The region is the second most densely populated in the country and contains 15.4% of Ghana's population with 5,055,883 inhabitants. 28 The capital city of the country, Accra, is located in this region.

### Data Sources and Data Cleaning

All data on COVID-19 cases recorded using three platforms sanctioned by the Ghana Health Service were used in this study. In the early stages of the outbreak, when the outbreak was limited to Greater Accra Region, data was entered into the Surveillance Outbreak Response Management & Analysis System (“SORMAS”, Helmholtz Centre for Infection Research. (2019). Braunschweig, Germany). However, as the outbreak continued to spread to other regions, additional platforms were introduced as a stopgap measure as the country expanded coverage of SORMAS beyond the epicenter. The Ghana Field Epidemiology and Laboratory Training Programme (GFELTP) together with the Geography department at the University of Ghana introduced the “ArcGIS” (Environmental Systems Research Institute (ESRI). (2014). ArcGIS online, Redlands, CA) and “Survey 123” (Environmental Systems Research Institute (ESRI) (2020), Survey 123, Redlands, CA) for collection of data on cases by epidemiologists and local health workers who were part of the response teams. Paper-based forms were also entered in REDCap. 30 This study pooled together the data from the three sources. The pooled data was cleaned using the unique identification numbers to remove duplicate entries. As part of the measure to protect patient identity, all personal identifying information were removed. The final cleaned dataset was used for this study.

### Data Collection

At the point of sample collection, patient characteristics were collected from the patients and entered into the various databases including the Surveillance Outbreak Response and Management and Analysis System (SORMAS), ArcGIS Survey 123, and hard copy case-based forms. Data on the hard copy case-based forms were later entered into REDCap. Information collected from patients included their sex, age, Region, district, education status, occupation, date of diagnosis, travel history (either from outside the country or district of diagnosis) point of entry for cases with travel history, whether there was previous contact with a confirmed case and symptoms status (symptomatic or asymptomatic). For symptomatic cases, data on the date of symptom onset was also collected. Geographical location coordinates were plotted for all identified cases. In determining the epidemiological link of cases, a description of the index case in each district was reviewed.

### Data Analysis

We conducted descriptive analysis by summarizing the data by person, place and time using median, frequencies, proportions, ratios, rates and maps. Categorical variables were expressed in frequencies and proportions. Continuous variables were summarized using measures of central tendency and their corresponding measures of dispersion. Age and sex distribution and ratios were reported. Case fatality rates were reported by region. Additionally, the geographical location of cases was used to build geospatial maps for the distribution of the cases using ArcGIS. We showed the epidemiological link between cases across the regions using index cases as points of movement. We reported local transmission and imported index cases from some regions to show the movement between and within the country.

An epidemiological curve was constructed for the outbreak. Further sub-group analysis was conducted showing the hotspots for COVID-19 infection at the initial two epicenters as at June 30, 2020 and the contact tracing outcomes for the first 30 cases recorded in the country. Denominators for some of the analysis varied due to missing data on some of the variables.

### Ethical Consideration

The Ghana Health Service collected data used in this manuscript as part of response activities for COVID – 19 pandemics in Ghana. We however sought approval from the Ghana Health Service to use the data (GHS-ERC 006/05/20). The confidentiality of data was maintained throughout the study. Data was kept on password protected computers and was only accesses by authorized persons.

## Results

### Characteristics of Cases (Person)

Out of approximately 300,000 tests conducted, 17,763 cases were detected as at June 30, 2020 with median age (IQR) of 33 (one month to 85 years). Majority of cases were males 10,272 (57.8%) with the age group of 20–29 years having the highest number of cases 3,075 (17.3%) (Table 1).

**Table 1:** Baseline Characteristics of COVID-19 Cases in Ghana, 2020

	Baseline Characteristics	Confirmed Cases N (%)
***Sex***		***n=17,763***
	Male	10,271 (57.8)
	Female	7491 (42.2)
***Age***		***n=10,487***
	0–9	250 (2.4)
	10–19	708 (6.8)
	20–29	3,073 (29.3)
	30–39	3,209 (30.6)
	40–49	1,652 (15.8)
	50–59	1,003 (9.6)
	>60	592 (5.6)
***Occupation***		***n=4,184***
	Health worker	683 (16.3)
	Formal employment	1,700 (40.6)
	Artisan/ Self employed	1,310 (31.4)
	Unemployed	491 (11.7)
***Symptoms Status***		***n=17,763***
	Symptomatic	3521 (19.8)
	Asymptomatic	14,242 (80.2)
***Common Symptoms***		***n=3,521***
	Cough	1,420 (40.3)
	Headache	1,092 (31.0)
	Fever	865 (24.5)
	General Weakness	667 (18.9)
	Chest Pain	300 (8.5)
	Joint/ Muscle Pain	248 (7.0)
	Sore throat	195 (5.5)
	Difficulty Breathing	130 (3.6)
***Period of diagnosis***		***n=17,763***
	Month One	1,648 (9.3)
	Month Two	5,545 (31.2)
	Month Three	7,380 (41.5)
	Month Four	3,190 (17.9)

*Denominators are different due to missing data for some of the variables

Among 4,184 respondents out of total confirmed cases, 1700 (40.6%) worked in the formal sector and 683(16.3%) were healthcare workers.

Among the confirmed cases, 3,521 (19.8%) were symptomatic of which cough was the highest symptom recorded in 1,420 (40.3%) cases. The remaining 14,242 (80.2%) confirmed cases were asymptomatic. Majority of the confirmed cases (7,380, [41.5%]) were detected in between May 2020 (Table 1).

### Location of Cases (Place)

The index cases for the country were detected on March 12, 2020 in Greater Accra region. Over 60% of the confirmed cases were reported in the Greater Accra Region (11,348 [63.9%]) followed by the Ashanti region (3,003 [16.9%]). By end of June, all 16 regions in the country had recorded cases of COVID-19. Out of 17,763 confirmed cases 13,190 (74.3%) had recovered as at June 30, 2020. Additionally, of the 4,573 active cases, Greater Accra Region had the highest number of active cases with 2,724. Overall case fatality rate for the country was 0.66% (Table 2)

**Table 2:** Recoveries and Active cases by Regions, Ghana, June 30, 2020

Date of recorded First Case	Region	Number of Cases N (%)	Recoveries/ Discharges N (%)	Number of Active Cases	Number of Deaths	CFR
**March 12, 2020**	Greater Accra	11,348 (63.9)	8624 (75.9)	2,724	53	0.47
**March 13, 2020**	Ashanti	3,003 (16.9)	1814 (60.4)	1,189	51	1.69
**March 14, 2020**	Western	741 (4.2)	691(93.3)	50	1	0.13
**March 18, 2020**	Eastern	773 (4.4)	507 (65.6)	266	2	0.26
**March 23, 2020**	Upper East	312 (1.8)	309 (99)	12	1	0.32
**March 25, 2020**	Upper West	38 (0.02)	38 (100)	0	0	0.00
**March 27, 2020**	Northern	56 (0.31)	26 (46.4)	30	2	3.57
**April 5, 2020**	Central	1,078 (6.1)	851 (78.9)	227	4	0.37
**April 5, 2020**	Volta	221(1.2)	199 (90)	22	1	0.45
**April 6, 2020**	North East	9 (0.05)	3 (33.3)	6	1	11.11
**April 17, 2020**	Oti	62 (0.34)	54 (87.1)	8	0	0.00
**April 17, 2020**	Western North	22 (0.12)	18 (81.8)	4	0	0.00
**May 15, 2020**	Savannah	34 (0.19)	31 (91.2)	3	0	0.00
**May 20, 2020**	Bono East	56 (0.31)	18 (32.1)	18	1	1.79
**May 25, 2020**	Ahafo	8 (0.05)	6 (75.0)	2	0	0.00
**May 29, 2020**	Bono	2 (0.01)	1 (50.0)	1	0	0.00
**Total**		17, 763	13, 190 (74.3)	4,573	117	0.66

### Regional Index Cases

The first index cases were reported in the Greater Accra Region. Both cases were imported cases from Turkey and Norway. The next three regions to record cases were the Ashanti, Central and Western Regions before the disease was recorded progressively in other regions. Of the sixteen regions, the index cases for 8 regions (50%) were local index cases. (Table 3)

**Table 3:** Epidemiological link of Regional Index COVID-19 cases in Ghana, June 2020

Date of Reported Index Case	Region	District Reporting Index Case	Places Visited Prior to detection (Region/ District/Country)	Remarks
**March 12, 2020**	Greater Accra	Ayawaso West	Norway, Turkey	Two imported cases were reported simultaneously.
**March 13, 2020**	Ashanti	Obuasi	UK	Two index cases detected. One is an imported case while the other is a resident (community transmission)
		Kumasi	Kumasi Resident	
**March 14, 2020**	Western	Shama	China	Imported
**March 18, 2020**	Eastern	Lower Manya Krobo	India	Imported
**March 23, 2020**	Upper East	Bolgatanga	Nkawkaw (Eastern Region)	Travelled between the two towns in a space of three days
**March 25, 2020**	Upper West	Wa	Greater Accra	Local transmission
**March 27, 2020**	Northern	Tamale	Guinea Residents – Travelled through Burkina Faso and Togo	Imported
**April 5, 2020**	Central	Cape Coast	UK, returnee	Imported
**April 5, 2020**	Volta	Aflao	Nigeria	Imported
		Hohoe	Greater Accra Region	Two index Cases who moved from Accra to Hohoe in a bid to escape lockdown restrictions
**April 6, 2020**	North East	West Mamprusi Municipal	Cargo Mate moving from Market to Market in the northern zone	Local Transmission. Posthumously confirmed.
**April 17, 2020**	Oti	Nkwanta	Greater Accra Region	Travelers to the Northern region who moved from Accra in order to avoid the mandatory lockdown announced by Government
**April 17, 2020**	Western North	Bibiani	Mines worker, No history of travel	Community Transmission
**May 15, 2020**	Savannah	East Gonja	Works in Salaga, Resident in Tamale (Northern Region) during weekends	Community transmission
**May 20, 2020**	Bono East	Tano South Municipal	Obuasi	Local Transmission (Moved from Ashanti Region – one Epicenter)
**May 25, 2020**	Ahafo	Asutifi South	Resident, No travel History	Community Transmission
**May 29, 2020**	Bono	Jaman North	Ivory Coast	Imported Case (Togolese National)

### Geo-Spatial Distribution of COVID-19 Cases

**Figure 1A-1D:**
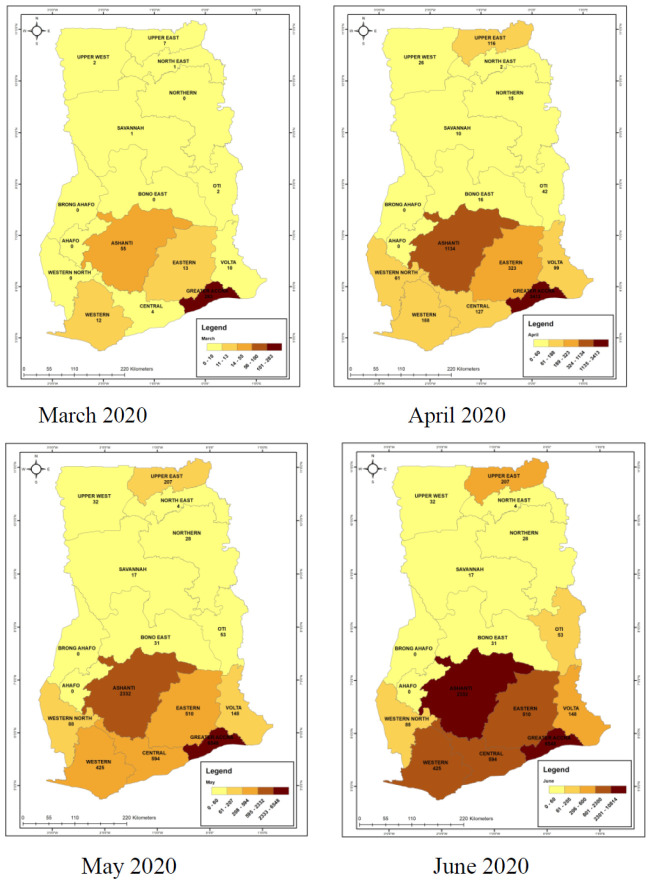
Cumulative Spatial maps showing geo-spatial distribution of COVID-19 Cases over the four-month period.

Over the past four months, the Geospatial maps of cumulative number of cases across the country showed that in March, few numbers of cases were reported in 4 regions in the country. From this initial four regions within month one, COVID-19 spread to fourteen out of the 16 administrative regions of the country by month two and by the third month, all sixteen regions had recorded cases with a general increasing cumulative trend (Figure 1A-D). The Greater Accra and Ashanti regions had the highest rate of increase in the number of cases with the number more than tripling in monthly intervals in Greater Accra Region.

### Hotspots for COVID-19 Infection at the two initial Epicenters

**Figure 2:**
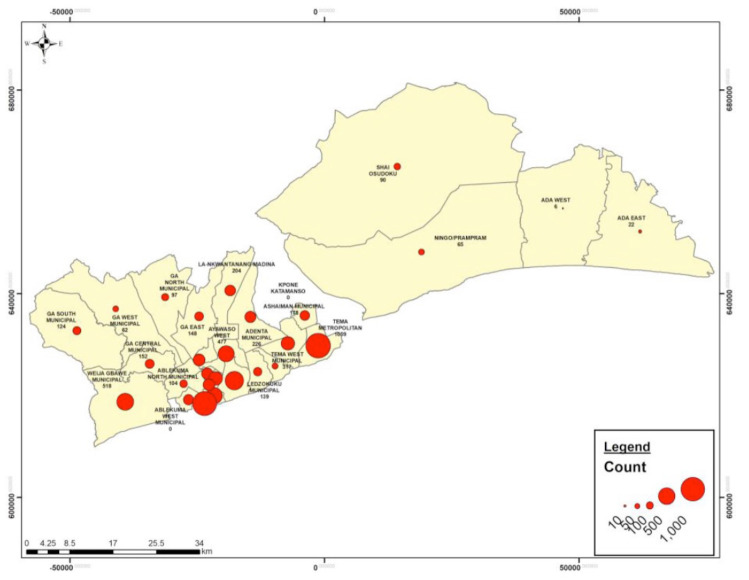
Dots Map showing the hotspots for COVID-19 in Greater Accra Region as at June 30^th^, 2020

Dots analysis showed that the hotspots for COVID-19 infection in Accra included the Accra Metropolis, Tema metropolis and Weija Gbawe municipal (Figure 2).

**Figure 3:**
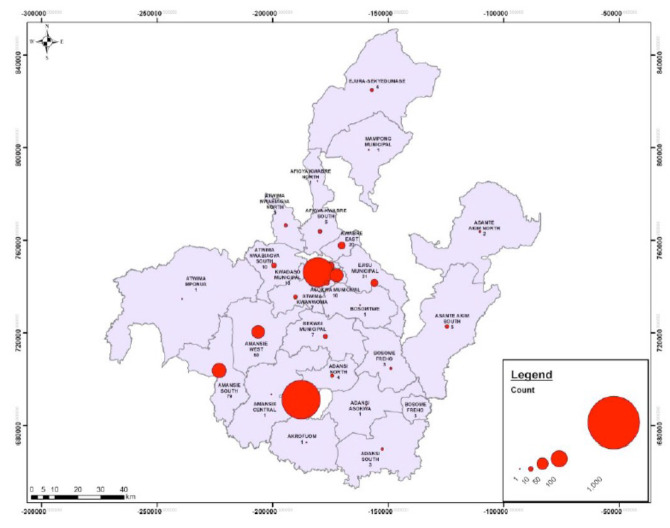
A dots map showing hotspots for COVID-19 infection in the Ashanti Region as at June 30^th^, 2020.

In Ashanti region, Obuasi municipal and Kumasi metropolitan were identified as hotspots with over 500 cumulative cases reported from these districts (Figure 3).

### Epidemiological Curve of COVID-19 Cases, March-June 2020

**Figure 4:**
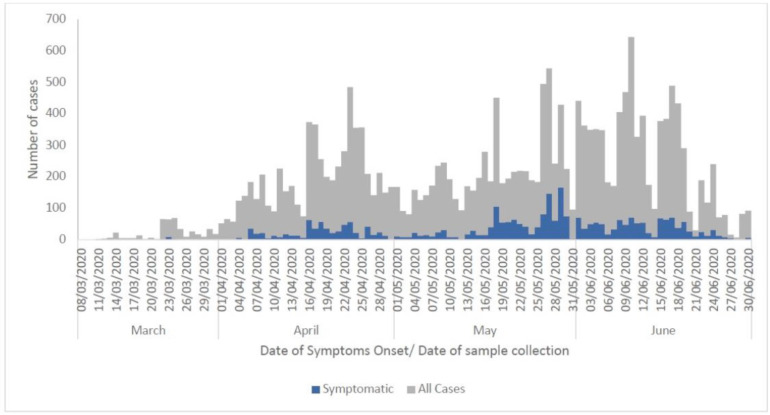
Epidemiological Curve of confirmed COVID-19 cases in Ghana, March 12, 2020 – June 30, 2020.

The distribution of cases showed a propagated outbreak with multiple peaks of about one-month interval. The highest peak of confirmed cases as at June 30, 2020 was observed at June 10, 2020. (Figure 4). The index cases were confirmed on March 12, 2020. Among symptomatic patients, cases were distributed over the four months with multiple peaks with the highest peak on April 29, 2020 (Figure 4).

### Contact Tracing for First Thirty Cases, Ghana

**Figure 5:**
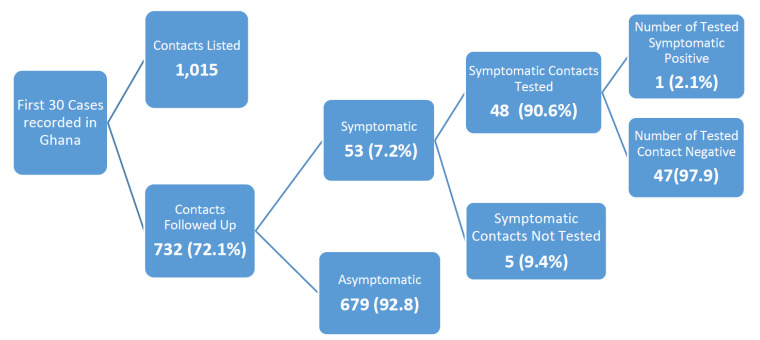
Contact Tracing for First 30 Cases of COVID-19 Outbreak, Ghana, 2020

From the first thirty cases recorded by the country, 1,015 contacts were listed of which 732 (72.1%) were successfully followed up. The number of contacts who developed symptoms and were tested were 53 with one contact testing positive for COVID – 19 as shown in Figure 5.

## Discussion

This study set out to determine the epidemiological characterization of the first 17,763 confirmed cases of COVID-19 that were recorded in Ghana from March 12 to June 30, 2020. Majority of the cases were males. This is in line with other epidemiological descriptive studies of COVID-19 where most cases were males as recorded in china and East Indonesia. 31,32 In a study conducted in Iceland, researchers were unsure if males being more affected may be due to more exposure or biologic tendencies.^33^ In similar epidemiological studies in China and Italy, findings showed that infected males were more likely to have severe disease though we could not establish or refute this finding in our setting due to unavailability of data on severity of cases. 34,35 However, this is an indication that gender is a risk factor for severity of COVID-19 and has a bearing on the gender specific mortality rate. In addition, this goes to support the global demographic statistics that men have a lower life expectancy than women. Although these findings were not established in Ghana, it gives an insight as to where we need to prioritize our gender specific interventions and risk communication. 36

In the first four months of the COVID-19 pandemic in Ghana, approximately 75% of recorded cases were asymptomatic. Having a large proportion of cases showing no symptoms has implications for control of the disease as cases who are unaware of their status will continue interacting with people thereby propagating the outbreak. Research has shown that large proportions of cases exhibit no symptoms especially in countries where screening is done amongst populations without symptoms.^37^–^40^ Ghana instituted an enhanced screening around radius of confirmed cases from March 31^st^, 2020 to April 27, 2020. Enhanced contact tracing is an advanced form of contact tracing which deals with the testing of samples from residents within certain specified area of an already confirmed case, with a goal of early case finding, isolation and control of the disease. A 1–2 km radius was used in Ghana's enhanced contact tracing basing on population density, social interaction within the localities and setting. Institution of enhanced contact tracing was a huge contributory factor to the high proportion of asymptomatic cases detected in the country as enhanced screening contributed over 63% of cases detected as at April, 2020 with 93% of cases detected through enhanced surveillance in the country being asymptomatic 41. In the USA, of 600 sailors aboard an aircraft that tested positive for COVID-19, over 60% had no symptoms and in homeless shelter in Boston, 100% of the 167 people who tested positive for COVID-19 were equally asymptomatic.^42^,^43^ These findings have been the driving force for advocation of masks wearing by the general population as a control measure.

Mandatory wearing of masks regardless of COVID-19 status is being implemented heavily in Ghana with support from the Government and all other institutions together with other preventive measures including regular hand washing. 41

The data shows that as demonstrated in other countries, COVID-19 is highly infectious and has spread into all 16 administrative regions of the country within the first 100 days of onset of the outbreak in the country. The epidemiological curves of all confirmed cases and symptomatic cases both show propagated outbreak with several peaks. The downward trends are not sustained long enough before another spike of cases is recorded. Although, the spread of the disease and numbers affected is slow in contrast to developed countries, the consistent increase in the peaks as the outbreak continues is a cause for concern. In the two main epicenters of the COVID-19 outbreak, identified hotspots were the Tema Metropolis and Weija Gbawe municipal in Accra. Tema metropolis is home to several industrial institutions and has recorded localized COVID-19 outbreaks in institutions including one in an inner food processing factory where over 600 cases were recorded. It is therefore not surprising that Tema metro is identified as a hotspot for COVID-19. Additionally, Accra metro is the central part of the capital where major trading activities take place. Looking at the prevalent practices that are associated with the metropolis including overcrowding, it is in line that the metro is identified as a hotspot as well. Kumasi Metropolis is also the central trading point in the Ashanti region. The identifications of these hotspots support the known epidemiological findings and underscores the need for heightened observation of preventive measures at social activities.

The epidemiological links between index cases also show that the disease spread to other regions through imported cases and some cases who moved out of the two epi-centers in a bid to avoid the lockdown that was instituted by the Government on March 30, 2020. 41 Our findings reiterate the need for timely response in the times of outbreaks of infectious diseases. Delays in implementing control measure bring about dire consequences and with the changing landscape of the COVID-19 outbreak in Ghana, there is a need for response efforts to be sustained in order to ensure control of the outbreak. As Ghana has begun to ease restrictions in the country with large numbers of students returning to school, and social events including churches, funerals and weddings opening up, we need to be mindful of a possible rebound of numbers of infected individuals and plan for it.

Details of Contact tracing that was conducted for the first thirty recorded cases showed that – only one contact tested positive amongst the 53 symptomatic contacts recorded. In the beginning of the outbreak, only symptomatic contacts were tested. This means that the country probably missed out on asymptomatic contacts who could have developed the infection as well. Several countries have recorded infections amongst asymptomatic contacts including china, USA and the United Kingdom (UK). 17,38–40 However, as the number of cases without travel history and having no epidemiological link to previous cases increased in Ghana, it became apparent that community level transmission was ongoing. This data was the basis for Ghana coming up with other strategies in case finding and response leading to the enhanced contact tracing strategy deployed in Ghana and this strategy led to a surge in the number of confirmed cases and with majority of them being asymptomatic.

As this study was purely based on routine data collected from detected cases, our study is limited by missing data. We compensated for this by using all available data sources in the country and cross validated the data retrieved from the sources. Additionally, we reported the exact number of verified data for each variable. Additionally, we were also limited by unavailability of data on important covariates such as comorbidities of the cases and the severity of the disease affecting our ability to draw conclusions on this.

## Conclusion

The epidemiological characteristics of the first 17, 763 COVID-19 cases in Ghana showed propagated spread of COVID – 19 detection in all 16 regions of the nation. More than a third of all cases detected nationwide were asymptomatic. Greater Accra and Ashanti regions have the highest number of recorded cases and active cases as of June 30th, 2020. Identified hotspot districts in Accra as Accra metropolis, Tema Metropolis and Weija-Gbawe municipal. In Ashanti Region, identified hotspots were the Obuasi municipal and Kumasi metropolitan areas. Continuous movement within the country at critical points after detection of the outbreak including days before lockdown fuelled the spread of the outbreak to other administrative regions.
